# Chloroquine analogs as antimalarial candidates with potent *in vitro* and *in vivo* activity

**DOI:** 10.1016/j.ijpddr.2018.10.002

**Published:** 2018-10-13

**Authors:** Anna C.C. Aguiar, Erika Murce, Wilian A. Cortopassi, Andre S. Pimentel, Maria M.F.S. Almeida, Daniele C.S. Barros, Jéssica S. Guedes, Mario R. Meneghetti, Antoniana U. Krettli

**Affiliations:** a Centro de Pesquisas Rene Rachou, Laboratório de Malária, Belo Horizonte, Brazil; b Pontifical Catholic University of Rio de Janeiro, Department of Chemistry, Rio de Janeiro, Brazil; c University of California, San Francisco, Department of Pharmaceutical Chemistry, USA; d Universidade Federal de Alagoas, Instituto de Química e Biotecnologia, Maceió, Brazil

**Keywords:** Malaria, Chloroquine, Resistance, Drug design

## Abstract

In spite of recent efforts to eradicate malaria in the world, this parasitic disease is still considered a major public health problem, with a total of 216 million cases of malaria and 445,000 deaths in 2016. Artemisinin-based combination therapies remain effective in most parts of the world, but recent cases of resistance in Southeast Asia have urged for novel approaches to treat malaria caused by *Plasmodium falciparum*. In this work, we present chloroquine analogs that exhibited high activity against sensitive and chloroquine-resistant *P. falciparum* blood parasites and were also active against *P. berghei* infected mice. Among the compounds tested, **DAQ**, a chloroquine analog with a more linear side chain, was shown to be the most active *in vitro* and *in vivo*, with low cytotoxicity, and therefore may serve as the basis for the development of more effective chloroquine analogs to aid malaria eradication.

## Highlights

• Resistance to recommended malaria therapies by WHO is emerging.
• Chloroquine analogs with modified side chains may overcome *P. falciparum* resistance.
• DAQ, a CQ-analog with a more linear side chain, is the most promising tested compound.
• DAQ may serve as a lead compound for the design of more effective antimalarials.

## Graphical abstract

Synthesis of *N*-(4-(dimethylamino)but-2-enyl)-7-chloro-quinolin-4-amine (**DAQ**): **DAQ**, a chloroquine analog with a more linear side chain, exhibited high activity against sensitive and chloroquine-resistant *P. falciparum* blood parasites and was also active against *P. berghei* infected mice.

**Figure:**
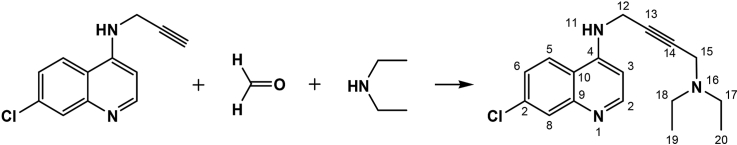

## 1 Introduction

Malaria remains a major public health problem and approximately 40% of the world population lives in areas of malarial endemicity distributed in 91 countries. The World Health Organization (WHO) reported a total of 216 million cases of malaria and 445,000 deaths in 2016, which represents an increase of 5 million cases over the previous year (WHO, 2017).

The early diagnosis and the successful drug treatment of infected patients are the main strategies for disease control. However, a recent rise in the artemisinin-based combination therapies (ACT) resistance against *Plasmodium falciparum* in Southeast Asia poses a serious threat to malaria control and its elimination globally, making the search for new antimalarial drugs urgent (Ariey et al., 2014; Talundzic et al., 2015).

Chloroquine (**CQ**), a 4-aminoquinoline drug, was extensively used worldwide in countries where malaria is endemic, being the most effective and the least expensive antimalarial for many decades, and is still recommended for treating *P. viva*x infections. Indeed, **CQ** has a rapid onset of action, low toxicity and is well tolerated (Wellems and Plowe, 2001). The most accepted and discussed mechanism of **CQ** is the inhibition of β-hematin formation in the digestive vacuole of the malaria parasite (Combrinck et al., 2013; Lehane et al., 2012; Olafson et al., 2015). However, the full understanding of CQ's mechanism is still controversial, and may include alteration of digestive food vacuole pH and inhibition of lactate dehydrogenase (Lehane et al., 2012; Read et al., 1999; Yeo et al., 2017). Even though there is a widespread resistance of *P. falciparum* and *P. vivax* to **CQ**, synthetic quinoline derivatives have remained a validated lead class for new drug discovery, since the resistance appears to be compound specific and not related to changes in the structure of the chloroquine targets (Hu et al., 2017; Lawrenson et al., 2018; Solomon et al., 2007). Even today, the quinoline core is still present in compounds in clinical trials such as ferroquine and in approved drugs like amodiaquine (Wells and Hooft van Huijsduijnen, 2015). Besides that, there is convincing evidence that significant and suitable structural changes on the side chain of the **CQ** molecule (either through altering its length or through the introduction of novel structural motifs) can circumvent **CQ**-resistance of the parasite (de Souza et al., 2014; Dola et al., 2017; Egan et al., 2000; Kaschula et al., 2002; Zishiri et al., 2011).

In the present work, CQ-analogs with different side chains were prepared and tested: (i) *in vitro* as blood schizonticides against both resistant and **CQ**-sensitive *P. falciparum* strains; (ii) and against *P. berghei* malaria in mice. We also evaluated: (iii) the cytotoxicity of the compounds; (iv) their ability to inhibit β-hematin formation; and (v) their binding mode to lactate dehydrogenase and dimeric hematin *in silico*.

## 2 Methods

### 2.1 Reagents and drug synthesis

All reactions for drug synthesis were performed under a 100% argon atmosphere using a dual vacuum/argon line and standard Schlenk techniques. Reagents and solvents were purchased from Sigma Aldrich and used without further purification. The IR spectra were recorded on a Varian 640-IR with an ATR device. The ^1^H NMR spectra were recorded at 400.130 MHz and the ^13^C NMR spectra at 100.613 MHz on a Bruker instrument (Bruker Avance 400) and were externally referenced to the tetramethylsilane (TMS). Chemical shifts (δ) and coupling constants (*J*) were expressed in ppm and Hz, respectively. High-resolution mass spectrometry (HRMS) was performed at the LTQ Orbitrap XL Hybrid Ion Trap-Orbitrap Mass Spectrometer by electrospray ionization (ESI) in positive mode. The melting or decomposition points of the isolated compounds were obtained at MSTecnopon instrument (PFMII Digital). Specific details about synthesis of compounds **PCQ**, **DAQ**, **CEQ** and **GIQ** are available in the Supporting Information.

### 2.2 Continuous cultures of *P. falciparum* and *in vitro* assays with *P. falciparum* infected erythrocytes

The activity of the CQ-analogs was evaluated against *P. falciparum* blood parasites [clone 3D7 a CQ-sensitive strain, and K1 a multidrug-resistant strain], which were cultured as previously described (Trager and Jensen, 2005). The freshly sorbitol synchronized ring stages were immediately incubated with the test compounds at various concentrations (from 10 to 0.152 μM or 1.0–0.0152 μM) that were previously solubilized in 0.05% dimethyl sulfoxide (DMSO) (v/v) (Lambros and Vanderberg, 1979). Each test was performed in triplicate in at least two different experiments. The results were compared with the control cultures in complete medium with no drugs. **CQ** was used in each experiment as an antimalarial control. The antiplasmodial activity of the compounds was measured through SYBR green assay (Smilkstein et al., 2004). Briefly, the plates were centrifuged at 700*g* for 5 min at room temperature to remove the medium, washed with PBS and incubated for 30 min with lysis buffer solution [2.4228 g TRIS, ultra-pure for 20 mM solution, pH 7.5; 1.8612 g of EDTA 5 mM ultrapure for 5 mM solution; 80 μg Saponin (0.008% w/v); 800 μL of Triton X-100 (0.08% v/v); water Type I] and SYBR green I DNA stain (1:20000). The fluorescence of uninfected erythrocytes was considered as background. Fluorescence was measured on fluorimeter (SpectraMax340PC384) at 485/535 nm.

The half-maximal drug inhibitory concentration (IC_50_) was estimated by curve fitting using the software from the OriginLab Corporation (USA) and compared to the parasite growth in the drug-free medium.

### 2.3 Cytotoxicity tests using immortalized cells

The cytotoxicity of CQ-analogs was evaluated in a human hepatoma cell line (HepG2) using cells cultured in 75-cm^2^ sterile flasks containing RPMI-1640 medium (supplemented with 10% heat-inactivated fetal bovine serum and 40 mg/L gentamicin) under a 5% CO_2_ atmosphere at 37 °C. When confluent, the cell monolayer was washed with culture medium, trypsinized, distributed in a flat-bottomed 96-well plate (5 × 10^3^ cells/well) and incubated for 18 h at 37 °C for cell adherence (Denizot and Lang, 1986). The compounds (20 μL), at various concentrations (400–1.0 μM), were placed in the 96-well plates, incubated with the cultured cells for 24 h under a 5% CO_2_ atmosphere at 37 °C and then the 3-(4,5-dimethylthiazol-2-yl)-2,5-diphenyltetrazolium bromide (MTT) solution (5 mg/mL; 20 μL/well for 3 h) was used to evaluate the mitochondrial viability. The supernatants were carefully removed and 100 μL DMSO were added to each well and mixed to solubilize the formazan crystals. The optical density was determined at 570 and 630 nm (background) (SpectraMax340PC384). The cell viability was expressed as the percentage of the control absorbance in the untreated cells after subtracting the appropriate background. Each test was performed in triplicate in at least two different experiments.

### 2.4 Inhibition of β-hematin formation assay

The assay was performed using a lipid as a catalyst to promote crystallization (Pisciotta et al., 2007; Fitch et al., 1999). Briefly, drug stock solutions were prepared in DMSO and were used at a final concentration of up to 30 mM. A heme stock (10 mM) was made in DMSO and was diluted to 50 μM with 100 mM sodium acetate (pH 4.8). A 10 mM 1-monooleoyl-rac-glycerol (MOG) stock was made in ethanol and was sonicated before a 50 μM heme stock was added to make 25 μM MOG–50 μM heme in 100 mM sodium acetate (pH 4.8). The 25 μM MOG–50 μM heme solution was sonicated and added to the assay plate at 100 μL/well. The plates were incubated at 37 °C for 2 h to allow crystallization, followed by the addition of 100 μL of 200 mM sodium bicarbonate (pH 9.1) to solubilize any remaining monomeric heme. After incubation for 30 min at room temperature, the amount of solubilized monomeric heme was determined by measuring the absorbance at 405 nm. Finally, 20 μL of 1 M sodium hydroxide were added to the plates to dissolve any crystals that had been formed. The absorbance was read at 405 nm to determine the total amount of heme present in each well. The inhibition of heme crystallization was determined as a function of the amount of monomeric heme that was not crystallized divided by the total amount of heme present in the assay mixture. The results are expressed as IC_50_ values based on the percentage inhibition of β-hematin formation by the compounds **GIQ**, **CEQ**, **PCQ** and **DAQ**. Each test was performed in triplicate in at least two different experiments.

### 2.5 *P. berghei* and antimalarial tests in mice

The suppressive test was performed as described (Peters, 1965). The *P. berghei* NK65 strain was obtained as a donation from New York University and maintained through weekly blood passages. For the experiments, groups of up to 30 mice were inoculated i.p. with 1 × 10^5^ infected erythrocytes, kept together for about 24 h, then randomly distributed into groups of five per cage. The mice were treated daily for three consecutive days with compounds freshly diluted in distillated water and administered orally at 50 mg/kg; the control groups received either the drug vehicle or the antimalarial **CQ** administered at 20 mg/kg. On days 5–15 after the parasite inoculation, blood was taken from the tail of each mouse and used to prepare thin smears that were methanol-fixed, Giemsa-stained, and examined microscopically (1000×) to determine parasitemia. The inhibition of parasite growth was determined in relation to parasitemia in the untreated mice, considered to be 100% parasite growth. Compounds reducing the parasitemia by >40% were considered active, between 30 and 40% partially active, and by less than 30% were considered inactive. The experiments were performed twice.

### 2.6 Docking studies

Compounds **GIQ**, **CEQ**, **PCQ**, and **DAQ** at different protonation states (Fig. S1) were minimized using a Monte Carlo approach with the software Ghemical (Hassinen and Peräkylä, 2001). Conformations with the lowest energy were chosen for further rigid docking calculations in dimeric heme and *P. falciparum* lactate dehydrogenase (*Pf*LDH) (PDB ID 1LDG) (Dunn et al., 1996). The choice for the structure of ferriprotoporphyrin IX followed the work of Casabianca and co-workers showing that chloroquine promotes the μ-oxo dimer formation in aqueous solution (Casabianca et al., 2008). The μ-oxo dimer was optimized using the Gaussian software v. 03, with the B3LYP functional, the 3-21G basis sets, and SFC = XQC to allow for convergence. Molegro Virtual Docker (MVD) was used for all docking calculations, as previously described, adding re-scoring energy functions for *Pf*LDH-inhibitor complexes (Aguiar et al., 2012; Cortopassi et al., 2011; Thomsen and Christensen, 2006). Similar docking approaches with heme have also been described by Saroj, Rajapakse and Dascombe and co-workers (Dascombe et al., 2005; Rajapakse et al., 2015; Verma et al., 2016). For protein-inhibitor docking, MVD internal charge scheme was considered and water molecules were conserved. **CQ** was chosen as a reference compound.

## 3 Results

### 3.1 Synthesis of CQ analogs

**Fig. 1:**
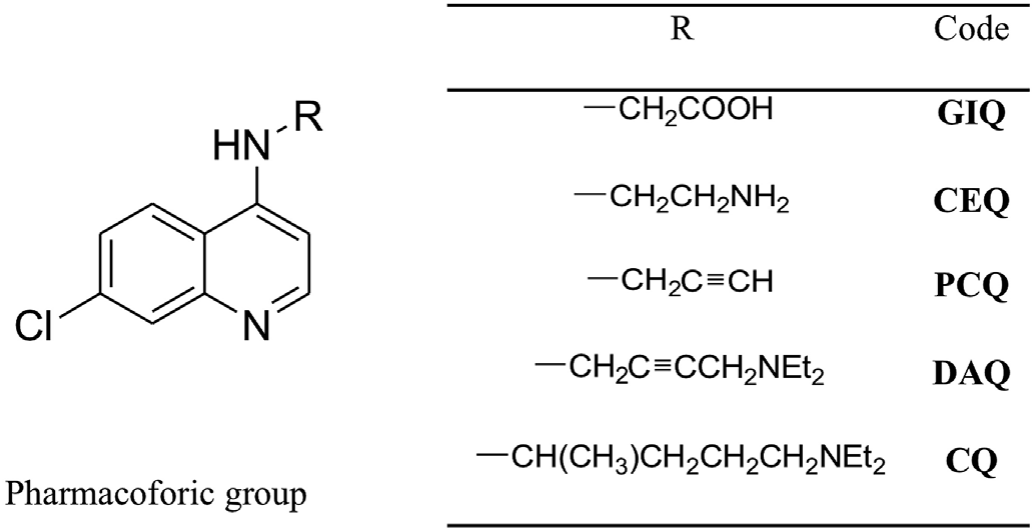
Molecular structure of the CQ derivatives.

In this study, we investigate the relationship between chemical structure and the antimalarial activity of CQ-analogs bearing different side chains. For that, we have prepared four CQ-analogs with different functional groups (see Supporting information) at the side chain, keeping three of the main points of the **CQ**'s molecular architecture: i) the quinoline ring and its substituents; ii) chloro and iii) amino group at the position 7 and 4 of the heterocyclic aromatic nucleus, respectively (see Fig. 1). These three structural features are believed to play an important role for **CQ**'s complexation with the toxic heme compound (ferriprotoporphyrin IX) formed during the digestion of the hemoglobin by the parasite and therefore inhibit the formation of the non-toxic hemozoin crystals (Gildenhuys et al., 2013; O'Neill et al., 2012). Indeed, the presence of the free heme moiety causes the death of the parasite. The **CQ**'s side chain is usually designed as a driving agent to increase the accumulation of the drug in the digestive vacuole of the parasite, whereas the presence of the pharmacophoric moiety, the quinoline ring, is important for the inhibition of hemozoin crystals formation (Bray et al., 2005; Manohar et al., 2010).

### 3.2 CQ-analogs are highly selective and active against resistant parasites

Four CQ-analogs were synthesized and tested against sensitive and resistant *P. falciparum* parasites *in vitro* (Table 1). The compounds **CEQ**, **PCQ** and **DAQ** were active against the sensitive (3D7 strain) and resistant (K1 strain) parasites at nanomolar dose, with IC_50_ ranging from 46 ± 4 to 405 ± 32 nM. The compound **GIQ** was inactive in all doses tested (highest dose 10 μM). **CQ** and **DAQ** were the most active compounds against the 3D7 sensitive strain with comparable IC_50_ values, however only **DAQ** was active against the resistant strain. The potency of **CEQ** and **PCQ** compounds was equivalent when compared to the susceptible and resistant strains. The selectivity index (SI, ratio between MDL_50_ and IC_50_) was determined using mammalian cells and the active compounds presented SI greater than 655, highlighting **DAQ** that demonstrated an SI almost 3 times higher than that found for **CQ** (Table 1).

**Table 1:** Antiplasmodial and cytotoxic activity of CQ-analogs.

Structure	IC_50_ (nM ± SD) *P. falciparum*		*MLD*_*50*_ (μM)	Selective index	
	3D7	K1	BGM-VN	3D7	K1
	>10000	>10000	>1000	Inactive	Inactive
	273 ± 12	218 ± 44	179 ± 40	655	821
	377 ± 14	405 ± 32	407 ± 185	1079	1005
	46 ± 4	50 ± 3	1481 ± 39	32195	29620
	36 ± 12	177 ± 20	420 ± 23	11666	2373

### 3.3 CQ-analogs are fast-acting inhibitors

**Fig. 2:**
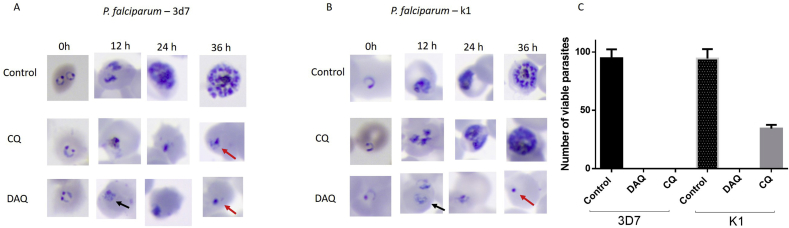
Microscopy of synchronized parasites continuously treated with **CQ** and **DAQ** at concentration of 10-fold the IC_50_ values and DMSO (control). Representative images of three independent experiments. (A) *P. falciparum* 3D7 **CQ**-sensitive parasite; (B) *P. falciparum* K1 **CQ**-resistant parasite. (C) Quantification of viable parasites by optical microscopy.

The time of inhibitory activity of the CQ-analogs was evaluated. The compounds were incubated at a concentration of 10-fold higher than IC_50_ values obtained for the *P. falciparum* 3D7 sensitive strain, with synchronized parasites. Then we observed the morphological changes by microscopy at 0, 12, 24 and 36 h post-synchronization (Fig. 2). The **CQ**-sensitive (3D7 strain) and resistant (K1 strain) parasites were tested in parallel, and the antimalarial **CQ** was used as a control. All CQ-analogs showed activity in the early ring stages against the **CQ**-sensitive 3D7 and **CQ**-resistant K1 parasites, inducing alterations of *P. falciparum* morphology, such as vacuolization (black arrow), between 0 and 12 h after incubation, and after 12 h of incubation picnotic nuclei were observed (red arrow), characterizing the fast time of action of the compounds. The drug **CQ** did not block the complete development of the parasites (Fig. 2c) in resistant line when the 10-fold IC_50_ for the sensitive line was applied. These data suggest a fast-acting mechanism in which the intraerythrocytic young forms of *P. falciparum* sensitive are susceptible to the effects of the compounds (Fig. 2).

### 3.4 CQ-analogs are active against *P. berghei* in mice

The compounds were administrated by oral route at 50 mg/kg during three consecutive days after infection; next, the parasitemia was checked until the day 15 and the animal's survival until day 30 post treatment. **CQ** was used as a positive control in the dose of 20 mg/kg. The compounds **DAQ** and **CEQ** were very active reducing the parasitemia 100% until the day 11 after infection and the mice survival in these groups was significantly higher (p < 0.05 by Mann-Whitney test) in comparison with the non-treated control. However, it was possible to observe a recrudescence of the parasitemia at day 11 after the treatment, and this phenomenon was more pronounced for the compound **PCQ.** Interestingly, these data corroborate with the *in vitro* findings, where **DAQ** and **CEQ** were the most active compounds. The compound **PCQ** was active reducing 70% the parasitemia on day 5 after infection. However, the animal's survival was not increased in relation to the untreated control. The animals treated with **CQ** showed no parasitemia until the last day of evaluation and survived until the last day of the experiment (Table 2).

**Table 2:** Antimalarial activity of CQ-analogs in mice infected with *P. berghei* after treatment with daily doses of the compounds during three consecutive days.

CQ-analog Dose mg/kg	Parasitemia on days^a^ (%reduction)						Survival
	5	7	9	11	13	15	
**CEQ**-50	0.00 (100)	0.00 (100)	0.00 (100)	0.00 (100)	0.57 (94)	4 (80)	26 ± 6^b^
**PCQ**-50	1.04 (73)	4.83 (10)	5.75 (8)	7.5 (32)	7.0 (32)	34.5 (0)	17 ± 8
**DAQ**-50	0.00 (100)	0.00 (100)	0.00 (100)	0.00 (100)	0.77 (92)	3.67 (82)	28 ± 1^b^
**CQ**-20	0.00 (100)	0.00 (100)	0.00 (100)	0.00 (100)	0.00 (100)	0.00 (100)	>30^b^
Non treated	3.84	5.42	6.24	10.8	10.4	20.2	19 ± 7

a Reductions ≤30% were considered as inactive, 30–50% as partially active and ≥50% as active drugs.

b Statistical differences by Mann-Whitney test were performed to compare treated and non-treated groups and are indicated by an asterisk (*p* < 0.05).

### 3.5 The antimalarial activity of CQ-analogs involves inhibition of β-hematin formation

Previous studies suggested a mechanism of action for CQ-analogs similar to the quinolinic antimalarials, *i.e.* they may act by inhibiting the formation of the hemozoin (Aguiar et al., 2012; Khan et al., 2009). Aiming to test if this model would also be valid for the CQ-analogs here evaluated, we performed the β-hematin formation *in vitro* assay and docking calculations of **GIG**, **CEQ**, **PCQ**, and **DAQ** to dimeric heme, and then compared to **CQ**.

The results showed that **DAQ** inhibited β-hematin formation with an IC_50_ value lower than **CQ**, whereas **CEQ** and **PCQ** inhibited β-hematin formation at concentrations 1.6 and 4-fold higher than that observed for **CQ** (Table 3). The compound **GIQ** was the least potent in the β-hematin formation as well as the least active *in vitro*.

**Table 3:** Inhibitory concentrations of β-hematin formation by CQ and analogs.

CQ-analog	β-hematin inhibition
	IC_50_ (mM)± SD
**GIQ**	7.6 ± 0.2
**CEQ**	1.2 ± 0.76
**PCQ**	3 ± 3
**DAQ**	0.15 ± 0.03
**CQ**	0.76 ± 0.46

**Fig. 3:**
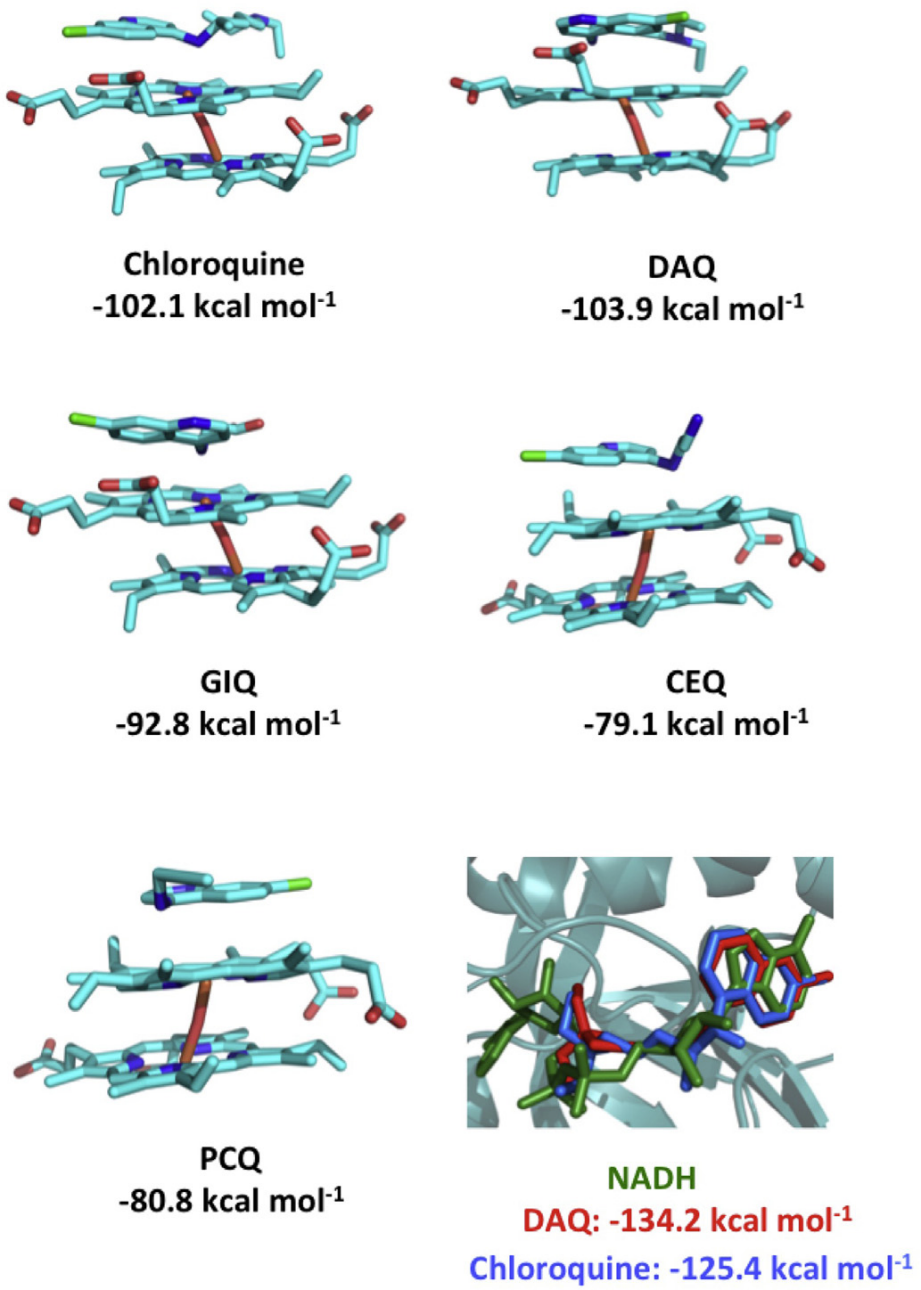
Docking results for **CQ** and its analogs **DAQ**, **GIQ**, **CEQ** and **PCQ** (top) to dimeric heme (bottom). At the bottom of the Figure, on the right, superimposed poses of **DAQ** (red) and **CQ** (blue) to the NADH (green) binding site in *Pf*LDH. (For interpretation of the references to colour in this figure legend, the reader is referred to the Web version of this article.)

Similarly, docking studies showed that these compounds were able to bind parallel to dimeric heme, as observed for **CQ** (Fig. 3). Only **DAQ** and **CQ** presented docking energies close to −100.0 kcal mol^−1^ (Fig. 3 and Figure S1). **DAQ** has high structural similarity to chloroquine, with a more linear structure due to the presence of a triple bond in its aliphatic chain. Interestingly, despite of these differences in the aliphatic chain, these compounds have similar docking poses (Fig. 3).

**CQ** is also shown to bind and inhibit *Pf*LDH, an enzyme that is crucial for the parasite survival by allowing the interconversion of lactate to pyruvate in late stages of glycolysis, using NADH as a cofactor (Menting et al., 1997; Read et al., 1999). Our docking results support a model where the **CQ** binds to the NADH site with low energies (−141.9 kcal mol^−1^), and the quinonilic ring of **CQ** superimposes the aromatic rings of this cofactor (Fig. 3), which led us to a model of inhibition by competition. Inhibitor-protein complexes were also built for **GIQ**, **CEQ**, **PCQ** and **DAQ**. We have observed that **DAQ** presented the lowest energy among the CQ-analogs (−134.2 kcal mol^−1^) and was also able to interact with the aromatic rings of NADH, which is suggestive of a similar mechanism to **CQ**. The other compounds **GIQ**, **CEQ** and **PCQ** presented docking energies higher than −114.0 kcal mol^−1^ (Fig. S1). Together, dimeric heme and *Pf*LDH docking results show **DAQ** as the most promising antimalarial compound among the molecules tested in this work, with predicted binding energies comparable to **CQ**, corroborating its high selectivity index (SI = 55,750) and low IC_50_ (0.1 mg/mL) for inhibition of β-hematin formation.

## 4 Discussion

The development of new CQ-analogs may help to overcome drug resistance, especially considering that it is believed to be stage specific and/or related to the compound structure (Gligorijevic et al., 2008; Stocks et al., 2002).

It is worth mentioning that the compound derived from the 4-aminoquinoline **CQ**, ferroquine (SSR97193, ferrochloroquine), currently in phase II of clinical development, has shown *in vitro* potential to overcome parasite resistance against **CQ** and other drugs (Atteke et al., 2003; Barends et al., 2007; Kreidenweiss et al., 2006). In addition, other antimalarial candidates, derived from **CQ**, have also shown potent antiplasmodial activity against **CQ**-resistant *P. falciparum* blood parasites (Kondaparla et al., 2017; Singh et al., 2016).

In the present work three compounds exhibited high activity against sensitive and **CQ**-resistant *P. falciparum* blood parasites, highlighting the activity demonstrated by **DAQ** that seems to have a mechanism to avoid the cross-resistance to **CQ**. Indeed, **DAQ** presented a higher SI than **CQ**. Besides, the compounds **DAQ**, **PCQ** and **CEQ** inhibited the *P. berghei* parasitemia *in mice*, translating the *in vitro* data. The high *in vivo* inhibition of **PCQ** and **DAQ** compounds is noteworthy. Briefly, this trend can be explained by the fact that the two most active compounds, **CQ** and **DAQ**, have the terminal amine functional group that can be protonated, allowing for a pH-trapping mechanism that increases their concentration in the digestive vacuole of the parasite. **GIQ** and **PCQ** miss this functional group and therefore are expected to be less active than **DAQ** and **CQ**.

Our *in silico* models suggest that **DAQ**, **PCQ** and **CEQ** are able to mimic **CQ**'s interactions with the dimeric heme, through a parallel complexation driven by *π*-π stacking with the quinolinic ring, a mechanism highlighted in recent literature for promising antiplasmodial candidates mimicking chloroquine (Dascombe et al., 2005; Rajapakse et al., 2015; Verma et al., 2016). Interestingly, **DAQ**, the compound with the highest SI, presented the closest energy values (−103.9 kcal mol^−1^) to **CQ**-heme complexation (−102.1 kcal mol^−1^). The structural change of the aliphatic chain of **CQ** to a more linear structure in **DAQ** does not affect its docking energy, and shows its ability to impair β-hematin formation by forming a heme-ligand complex known to be toxic to the parasite. Another mechanism of action for chloroquine has also been suggested, highlighting its potential as a weak inhibitor of *Pf*LDH, through competition with the NADH active site (Menting et al., 1997; Read et al., 1999). **DAQ** is shown to have the lowest energy interaction with *Pf*LDH among all tested CQ-analogs (−134.2 kcal mol^−1^), and is also able to interact with the NADH binding site, corroborating our *in vitro* and *in vivo* data suggesting **DAQ** as the most promising **CQ** inhibitor among the three analogs tested in this work.

## Conflicts of interest

The authors of this manuscript have no conflict of interests.
