# Supplementary material for: Chloroquine analogs as antimalarial candidates with potent in vitro and in vivo activity
Source: Int J Parasitol Drugs Drug Resist. 2018 Oct 13;8(3):459–64. doi: 10.1016/j.ijpddr.2018.10.002 (PMC6215995; doi:10.1016/j.ijpddr.2018.10.002)
Supplement: Supporting Information [file mmc1.docx]

**
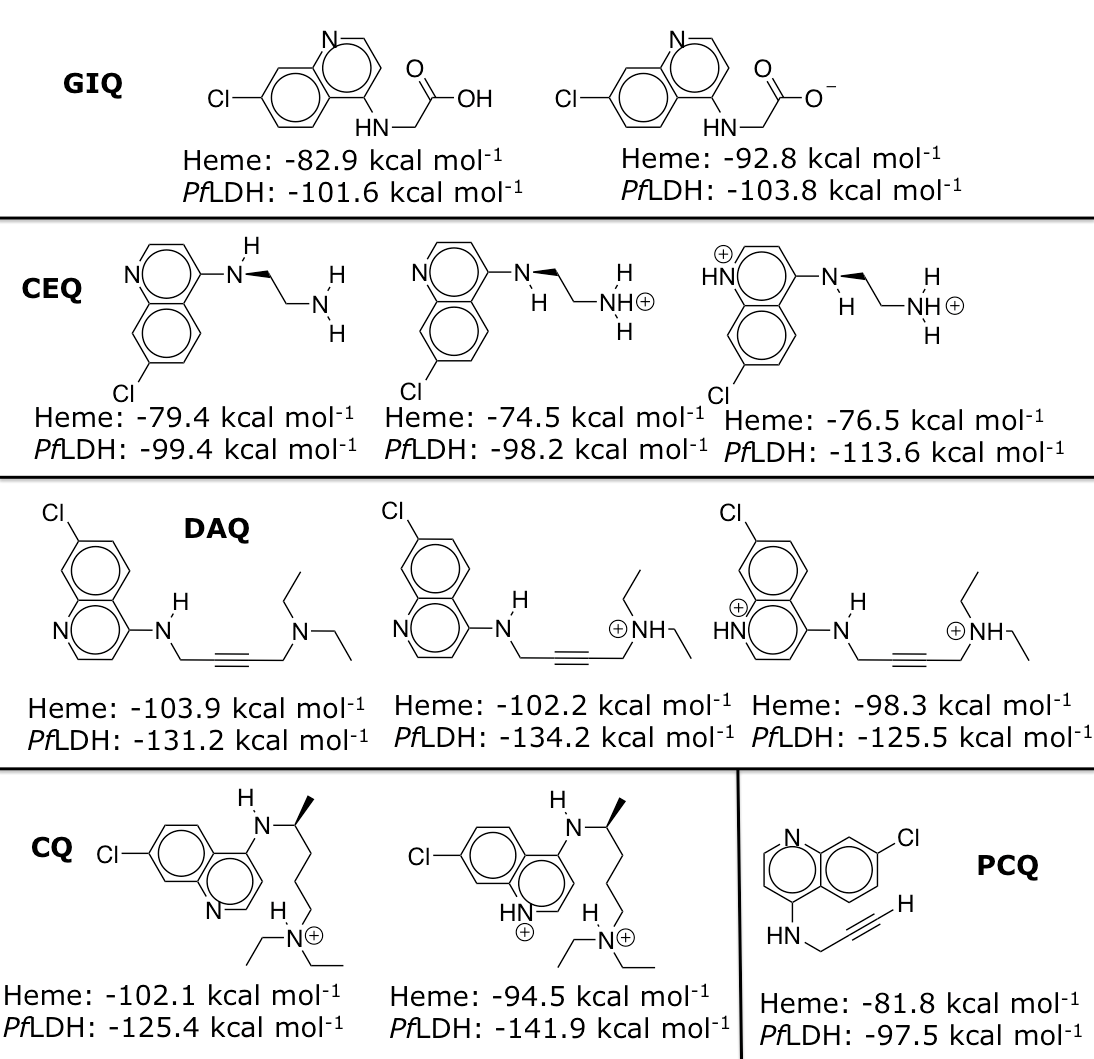
**

**Figure S1**. Docking energies for different protonation states of **CQ** and its analogs **DAQ**, **GIQ**, **CEQ** and **PCQ** to dimeric heme and *Pf*LDH.

**Synthesis of *N*-(prop-2-enyl)-7-chloroquinoline-4-amine** (**PCQ**)

For the synthesis of **PCQ** we adapted methodologies based on the work of Thi and Fisher and co-workers.(Fisher et al., 2014; Nguyen et al., 2008) Briefly, in a sealed 20 mL-tube it was added 4,7-dichloroquinoline (1.5 g, 7.6 mmol) and phenol (7.15 g, 76 mmol). After 1 h under stirring at 100 °C, propargylamine (0.972 mL, 15.2 mmol) was added and the temperature was raised to 120 °C, leaving under stirring for a further 4 h. At the end of the period, the mixture was cooled to room temperature and aqueous solution 15% NaOH (30 mL) was added to the reaction mixture. There was formation of a yellow solid, which was washed with more NaOH solution and then with water. The solid obtained was purified by column chromatography using as eluent a mixture of hexane: acetate in the proportion of 6: 4 and silica-gel. After elimination of the volatiles, the product was obtained as a yellow solid. Yield: 92%.

Molecular Mass: 216.67 g/mol; HRMS-ESI for C_12_H_9_ClN_2_ (m/z): calculated 217.0527 (M + H^+^); found 217.0525 (M + H^+^). Decomposition Point: 238.1 ºC.

^1^H NMR (400 MHz, MeOD): δ 2,66 (t, *J* = 2.45 Hz, 2H, 14-CH), 3.44 (s, 1H, N-H aminic), 4.18 (d, *J* = 2.45 Hz, 2H, 12-CH_2_), 6.67 (d, *J* = 5.59 Hz, 1H, 3-Ar-H), 7.43 (dd, *J* = 2,14 Hz and *J* = 8.91 Hz, 1H, 6-Ar-H), 7.81(d, *J* = 2.22 Hz, 1H, 8-Ar-H), 8,04 (d, *J* = 8.91 Hz, 1H, 5-Ar-H), 8.43(d, *J* = 5.58 Hz, 1H, 2-Ar-H).

^13^C NMR (100 MHz, MeOD-d_4_): δ 33.01 (C-12), 73.01 (C-14), 80.42 (C-13), 100.99 (C-3), 119.09 (C-10), 124.43 (C-5), 126.57 (C-6), 127.77 (C-8), 136.65 (C-7), 149.60 (C-9), 152.23 (C-2) and 152.46 (C-4).

I.R. (ν_máx_/cm^−1^): 3291 (ν ≡CH), 3214 (ν N-H), 3062 (ν =CH), 2921 (ν_as_ CH_2_), 2859 (ν_s_ CH_2_), 2161 (ν C≡C), 1581 (ν C=C), 1448 (δ CH_2_), 1320 (C_AR_-N), 1280 (C_R_-N), 846 (δ =CH) and 809 (δ =CH).

**Synthesis of *N*-(4-(dimethylamino)but-2-enyl)-7-chloro-quinolin-4-amine (DAQ**)

For the synthesis of **DAQ** we used a methodology adapted from that employed by Ebeling and Singh and co-workers.(Ebeling et al., 2002; Singh et al., 1969) In a 50 mL-round bottom flask it was added an aqueous solution of 50% diethylamine (3.92 mL, 18.55 mmol) and paraformaldehyde (0.450 mL, 6.01 mmol). The mixture was stirred for 1 h and added into another 50 mL- round bottom flask containing **PCQ** (1 g, 4.62 mmol), CuI (0.01158 g, 0.060 mmol) and ethanol (20 mL) which was left under reflux for 24 h. The mixture was then filtered and the liquid was dried in a rotatory-evaporator. The crude solid was recrystallized with ethanol/water, recuperated by filtration and dried under vacuum, leading to a light yellow solid. Yield: 40%.

Molecular Mass: 301.13 g/mol; HRMS-ESI for C_17_H_20_ClN_3_ (m/z): calculated 302.1419 (M + H^+^); found 302.1421 (M + H^+^). Melting point: 153.8 ºC.

^1^H NMR (400 MHz, MeOD-d_4_): δ 1.01 (t, 6H, *J* = 7.21 Hz, 19 e 20-CH_3_), 2.52 (q, 4H, *J* = 7.23, 17- and 18-CH_2_), 3.41 (t, 2H, *J* = 1.91 Hz, 15-CH_2_), 4.21 (t, 2H, *J* = 1.91 Hz, 12-CH_2_), 6.69 (d, 1H, *J* = 5.62 Hz, 3-CH), 7.43 (dd, 1H, *J* = 2.15 e 9.03 Hz, 6-CH), 7.81 (d, 1H, *J* = 2.12 Hz, 5-CH), 8.04 (d, 1H, *J* = 9.01 Hz, 5-CH) and 8.43 (d, 1H, *J* = 5.53 Hz, 2-CH).

^13^C NMR (100 MHz, MeOD-d_4_): δ 12.19 (C-19 e C-20), 33.19 (C-12), 41.39 (C-15), 48.23 (C-17 e C-18), 78.14 (C-14), 82.36 (C-13), 101.19 (C-3), 119.12 (C-10), 124.41 (C-5), 126.53 (C-6), 127.79 (C-8), 136.61 (C-7), 149.61 (C-9), 152.22 (C-2) and 152.37 (C-4).

I.R. (ν_máx_/cm^−1^): 3237 (ν N-H), 3068 (ν =CH), 2965 e 2859 (ν_as_CH_3_ e ν_s_CH_3_), 2933 and 2815 (ν_as_CH_2_ and ν_s_CH_2_), 2161 (ν C≡C), 1579 (ν C=C), 1442 (δ CH_2_), 1367 (δ CH_3_), 1311 (C_AR_-N) and 1238 (C_R_-N).

**Synthesis of *N*-(2-aminoethyl)-7-chloroquinolin-4-amine** (**CEQ**)

The **CEQ** was prepared according to the methodology adapted from Iwaniuk, Rudrapal and co-workers.(Iwaniuk et al., 2009; Rudrapal et al., 2013) In a 50 mL-round bottom flask were introduced 4,7-dichloroquinoline (1.0 g, 5.0 mmol) and ethylenediamine (3.35 mL, 50 mmol). This mixture was stirred under reflux for 6 hours. The reaction mixture was cooled to room temperature and basified with NaOH(aq) 10% (3 mL). Immediately, the formation of a yellow solid is observed. This solid was washed with NaOH(aq) 10% (3x, 3 mL) and then with distilled water (3x, 5 mL). The yellow solid was finally dried under vacuum. Yield: 72%.

Molecular Mass: 221.69 g/mol; HRMS-ESI for C_11_H_12_ClN_3_ (m/z): calculated 222.0793 (M + H^+^); found 222.0791 (M + H^+^). Melting point: 148.5 ºC.

^1^H NMR (400 MHz, MeOD-d_4_): δ 2.96 (t, 2H, *J* = 6.41 Hz, 13-CH_2_), 3.44 (t, 2H, *J* = 6.42, 12-CH_2_), 6.56 (d, 1H, *J* = 5.66 Hz, 3-CH), 7.40 (dd, 1H, *J* = 2.21 and 9.00 Hz, 6-CH), 7.77 (d, 1H, *J* = 2.15 Hz, 8-CH), 8.12 (d, 1H, *J* = 9.00 Hz, 5-CH) and 8.35 (d, 1H, *J* = 5.56 Hz, 2-CH).

^13^C NMR (100 MHz, MeOD-d_4_): δ 40.96 (C-13), 46.45 (C-12), 99.84 (C-3), 118.95 (C-10), 124.50 (C-5), 126.18 (C-6), 127.74 (C-8), 136.49 (C-7), 149.81 (C-9), 152.61 (C-2) and 152.98 (C-4).

I.R (ν_máx_/cm^−1^): 3303 (ν NH_2_), 2925 (ν_as_CH_2_), 2861 (ν_s_CH_2_) and 1583 (ν C=C), 1544 (δ N-H), 1454 (δ_s_ CH_2_) and 1322 (ω CH_2_).

**Synthesis of Acid 2- (7-chloroquinolin-4-ylamino) acetic (GIQ**)

For the preparation of **GIQ** we used methodologies adapted from these used by Starcevik and co-workers.(Starcevic et al., 2012) Briefly, in a 50 mL-round bottom flask we added 4,7-dichloroquinoline (1.98 g, 10 mmol), glycine (1.50 g; 20 mmol), and phenol (5.42 g, 57.6 mmol). The mixture was kept under reflux and stirring for 18 hours. After this period, an additional 1 eq. of glycine (0.750 g; 10 mmol) was added. The reaction mixture was stirred for 3 hours and then cooled to room temperature. Then, ethyl acetate (22 mL) was added to the mixture. The solid was recuperated by filtration, washed with ethyl acetate, and then dissolved in hot 10% Na_2_CO_3_ (10 mL). The solution was cooled to 5 °C and the pH was adjusted to 6 (with a 6 M HCl solution). The solid formed was recuperated by filtration, washed with distilled water and toluene and dried in a high vacuum. At this stage, a light gray solid was obtained. Yield: 42%.

Molecular Mass: 236.65 g/mol; HRMS-ESI for C_11_H_9_ClN_2_O_2_ (m/z): calculated 237.0425 (M + H^+^); found 237.0421 (M + H^+^). Decomposition point: 287.9 ºC.

^1^H NMR (400 MHz, MeOD-d_4_): δ 4.04 (d, 2H, *J* = 6.05 Hz, 12-CH_2_), 6.36 (d, 1H, *J* = 5.45, 3-CH), 7.49 (dd, 1H, *J* = 2.22 and 9.00 Hz, 6-CH), 7.81 (d, 1H, *J* = 2.24, 8-CH), 8.22 (d, 1H, *J* = 9.01 Hz, 5-CH) and 8.40 (d, 1H, *J* = 5.44 Hz, 2-CH).

^13^C NMR (100 MHz, DMSO-d_6_): δ 44.80 (C-12; CH_2_), 99.67 (C-3), 117.72 (C-10), 124.43 (C-5), 125.01 (C-6), 127.42 (C-8), 134.22 (C-7), 148.72 (C-9), 150.89 (C-2), 151.71 (C-4) and 171.70 (C-13; C=O).

I.R. (ν_máx_/cm^−1^): 3559 (ν O-H), 2911 (ν_as_CH_2_), 2805 (ν_s_CH_2_), ≈ 1650 (C=C e C=O) and 905 (δ OH).
